# Primary care management of Long-Term opioid therapy

**DOI:** 10.1080/07853890.2022.2121417

**Published:** 2022-09-16

**Authors:** Phillip O. Coffin, Rebecca S. Martinez, Brian Wylie, Bunny Ryder

**Affiliations:** San Francisco Department of Public Health, Center on Substance Use and Health, San Francisco, CA, USA

**Keywords:** Opioids, chronic pain, opioid stewardship, naloxone, substance use disorder, buprenorphine

## Abstract

The United States underwent massive expansion in opioid prescribing from 1990–2010, followed by opioid stewardship initiatives and reduced prescribing. Opioids are no longer considered first-line therapy for most chronic pain conditions and clinicians should first seek alternatives in most circumstances. Patients who have been treated with opioids long-term should be managed differently, sometimes even continued on opioids due to physiologic changes wrought by long-term opioid therapy and documented risks of discontinuation. When providing long-term opioid therapy, clinicians should document opioid stewardship measures, including assessments, consents, medication reconciliation, and offering naloxone, along with the rationale to continue opioid therapy. Clinicians should screen regularly for opioid use disorder and arrange for or directly provide treatment. In particular, buprenorphine can be highly useful for co-morbid pain and opioid use disorder. Addressing other substance use disorders, as well as preventive health related to substance use, should be a priority in patients with opioid use disorder. Patient-centered practices, such as shared decision-making and attending to related facets of a patient’s life that influence health outcomes, should be implemented at all points of care.

**Key messages**
- Although opioids are no longer considered first-line therapy for most chronic pain, management of patients already taking long-term opioid therapy must be individualised.
- Documentation of opioid stewardship measures can help to organise opioid prescribing and protect clinicians from regulatory scrutiny.
- Management of resultant opioid use disorder should include provision of medications, most often buprenorphine, and several additional screening and preventive measures.

## Introduction

Managing opioids in primary care today is complicated. Starting in the 1990s, healthcare went through a period of vast expansion of opioid prescribing, contributing an an epidemic of opioid overdose deaths from prescribed opioids, and subsequent waves of the overdose crisis [1]. Beginning in 2010, and accelerating in 2016, the pendulum swung back, with increasing restrictions on opioid prescribing. The Drug Enforcement Administration (DEA) shuttered multiple clinics that overprescribed and dispensed vast amounts of opioid medications [2]. Numerous interventions were implemented including controlled substance monitoring programs (CSMPs, commonly referred to as “Prescription Drug Monitoring Programs” or “PDMPs”, but almost exclusively tracking only controlled substances), controlled substance patient-clinician agreements, urine drug screening, co-prescribing of naloxone, and opioid dose and duration restrictions. In many cases, clinicians are legally required to utilise these tools when prescribing opioids. Investigations weren’t limited to just the DEA, but also medical boards [3]. Clinicians became both concerned and overwhelmed; many now refuse to provide opioid medications at all, or believe they should cease prescribing opioids to patients who have received prescription opioids for years.

In this review, we provide guidance for primary care clinicians on initiating, continuing, and tapering opioid medications; following expected opioid stewardship guidelines; and managing opioid use disorder. We attempt to balance the competing demands of primary care practice with patient safety and well-being.

## Methods

This invited manuscript represents the outcome of six years of iterative work developing trainings in opioid management for clinicians. The authors include clinicians with extensive primary care experience, expertise in addiction medicine, and experience developing opioid management guidelines. The concepts and materials in this review have been previously summarised in *Opioids and Chronic Pain: A Guide for Primary Care Providers*, available at www.ciaosf.org/materials.

*Opioids and Chronic Pain: A Guide for Primary Care* Providers is a curriculum for academic detailing on opioid management in primary care that was developed by the authors based on national, state, and professional society guidelines, published research, and clinical experience, with review by external experts in primary care, addiction medicine, and pain management. This curriculum has been updated at least every two years (latest update April 2022). While the information included does not conflict with the guidelines of the Centres for Disease Control and Prevention (CDC) [4], the recommended approaches are more closely aligned with the revised guidelines under public comment during the preparation of this manuscript [5]. This manuscript represents the opinions of the authors, should not be considered as clinical guidelines, and should not replace clinical judgement.

## Using opioids for chronic pain

### Management of chronic pain

Chronic pain is a complex and multifaceted set of disorders, ranging from lower back pain, hip osteoarthritis, and bony infarcts from sickle cell disease to fibromyalgia, migraine headaches, and peripheral neuropathy. Assessment of chronic pain should include a full history and physical, including descriptions of the pain condition, as well as the impact of pain on physical and social functioning. The mechanism of pain should be identified (e.g. neuropathic, musculoskeletal, inflammatory, multimodal) and used to guide therapies. Treatment offerings should include both non-pharmacologic and pharmacologic options (see Table 1).

**Table 1.** Options for chronic pain management.

*Medications*	Integrative therapies	Behavioural health	Movement-based therapies	Procedural interventions
*NSAIDs / acetaminophen*	Manual medicine	Group therapy for pain management	Physical therapy	Ice or heat
*Anticonvulsants*	Chiropractic	Therapy for anxiety or depression	Occupational therapy	Local injections
*Antidepressants*	Acupuncture	Social engagement plan	Supervised physical activity	Transcutaneous electrical nerve stimulation
*Topical therapies*	Herbs and supplements	Cognitive behavioural therapy	Graded physical activity	Low-level laser therapy
*Immunomodulators*	Yoga, Tai Chi, or mindful movement	Acceptance and Commitment Therapy	Multidisciplinary rehabilitation	Monopolar dialectic radiofrequency
*Muscle relaxants*	Massage			Surgical interventions
*Cannabinoids*	Alexander Technique			
*Opioids*				

**Figure 1.**
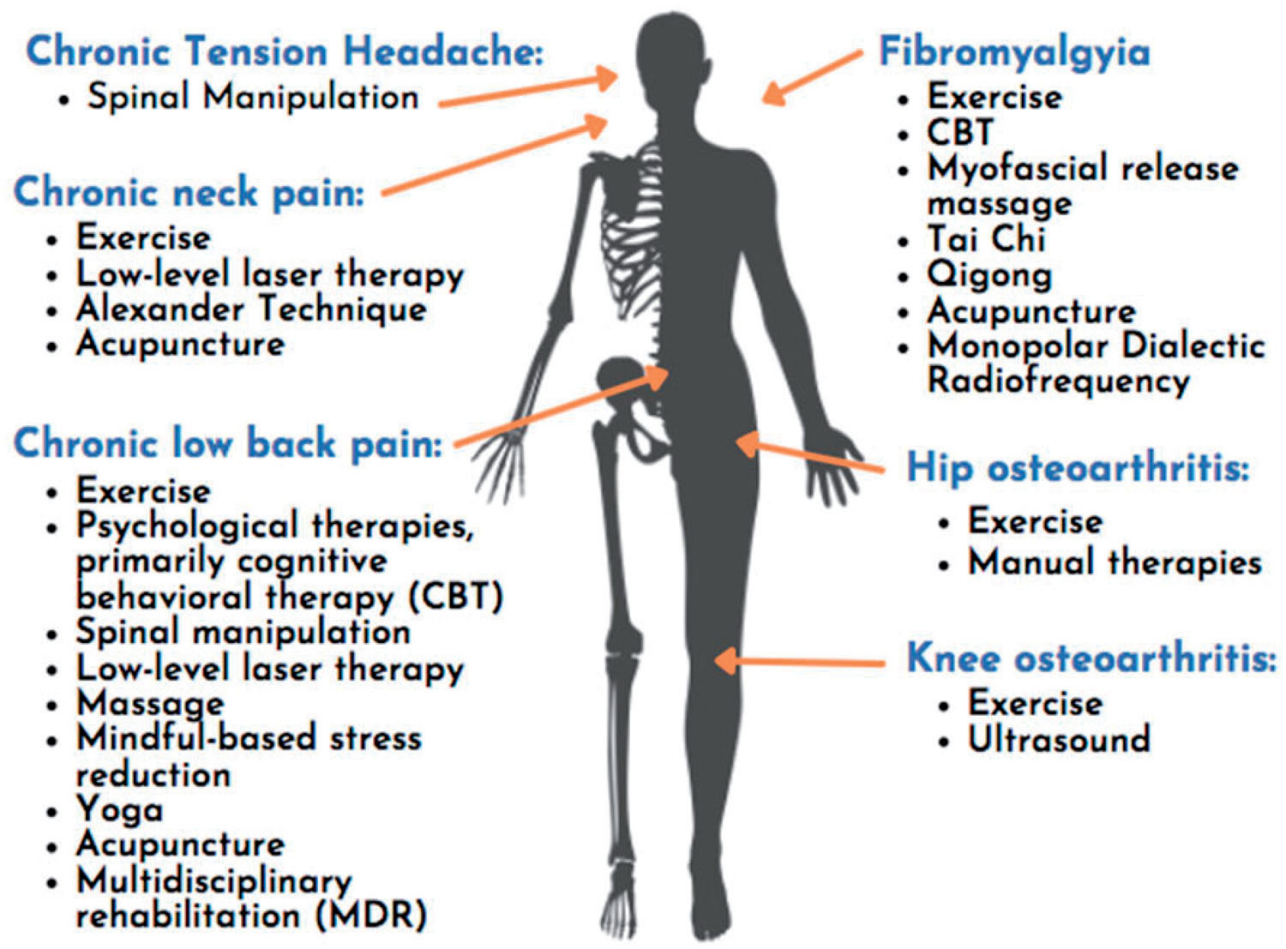
Interventions with evidence of benefit for selected chronic pain conditions [6]. Adapted from the authors’ public domain document “Opioids and Chronic Pain: A guide for primary care clinicians”, San Francisco Department of Public Health, accessed at www.ciaosf.org on 30 November 2021.

Non-pharmacologic interventions include movement-based therapies, integrative therapies, behavioural therapies, and procedures. The Agency for Healthcare Research and Quality (AHRQ) conducted a systematic review of non-pharmacologic interventions and found multiple interventions for six common chronic pain conditions (see Figure 1) [6].

Medications commonly used in the treatment of chronic pain include non-steroidal anti-inflammatory agents (NSAIDs) and acetaminophen, anticonvulsants, antidepressants, topical therapies, immunomodulators (DMARDs), muscle relaxants, cannabinoids, and opioids. The AHRQ conducted a similar systematic review of non-opioid medications for chronic pain, however the results are limited to medications that have been studied for these conditions, and subject to the biases of pharmaceutical manufacturer research investment [7]. Thus, most guidelines rely upon expert opinion in considering medications for pain conditions.

Opioid medications should be avoided as first-line treatment for chronic pain, with certain exceptions. First, some conditions (e.g. sickle cell pain crises) are often best managed by opioid medications in conjunction with non-opioid interventions [8]. In addition, pain due to metastatic cancer or other end-of-life conditions often may be best managed by opioids. In these circumstances, opioids are selected because pain tends to worsen over time, requiring escalating doses, while other failing organ systems may limit the use of alternative medications for pain. In addition, in the setting of end-of-life care there is less concern regarding the development and sequelae of opioid use disorder (OUD), and the benefits of opioid therapy outweigh the risks in most cases.

In addition, opioid therapy, particularly when provided in low-dose or for intermittent use, may be appropriate for patients with other pain conditions who are not candidates for non-opioid pain management approaches (e.g. when other medications are contraindicated). A key study in the use of opioids for chronic pain was conducted by Krebs, et al. [9] The investigators randomised 240 patients with chronic moderate-to-severe back, hip, or knee osteoarthritis to either opioids or non-opioid medications. Over 12 months of care no significant differences in pain-related function were noted. Pain intensity was reported less in the non-opioid group and more adverse medication-related symptoms occurred in the opioid group. While this study suggests that non-opioid medications are likely superior for these conditions, an important caveat is that the study did not include patients with any contraindications to NSAIDs or acetaminophen (e.g. history of gastrointestinal bleeds, or impaired renal or hepatic function) or patients who had been on opioids previously. Given the absence of other established medications for these conditions, this study does not provide information on how to manage patients who were already using opioid medications, nor those with contraindications to NSAIDs and acetaminophen. Limiting opioids for these conditions, when possible, to low or intermittent dosing helps to mitigate risks and the development of tolerance associated with high-dose opioid therapy, while reducing the quantity of opioids potentially available to the broader community due to diversion [10].

#### Starting opioid therapy

Most guidelines and experts agree that short-acting opioids are preferred to long-acting formulations when initiating opioid therapy for opioid naïve patients [4]. However, dosing considerations depend heavily on the indication for treatment. For pain associated with metastatic cancer, for example, treatment with opioids may begin and be titrated upward rather rapidly. In contrast, if opioid therapy is utilised to treat a condition such as hip or knee osteoarthritis in the context of inability to tolerate alternative therapies, the clinician and patient should strive to minimise the dose and aim for intermittent use.

The CDC recommends reassessing the risks and benefits of opioid therapy as dose approaches 50 morphine milligram equivalents (MME), and avoiding doses over 90 MME unless they can be justified [4]. These dose thresholds were selected based on data indicating opioid overdose risk increases somewhat linearly with dose [11]. Dose thresholds have since been integrated extensively into standards-of-care and used as metrics of success for health plans, clinics, and individual clinicians. These standards have resulted in problematic patient outcome associated with sudden discontinuation and forced tapers of high-dose opioid therapy [12]. The CDC and the Food and Drug Administration (FDA) have clarified that the dose limit recommendations were intended for opioid-naïve patients and thus should not be applied to patients already receiving high-dose opioids [12]. A recent CDC panel recommended eliminating opioid dose thresholds due to the unintended consequences of rapid tapers and opioid discontinuations (e.g. transitions to non-prescribed opioids, overdose, mental health crises, and suicide) that have affected and harmed many patients who were already receiving high-dose opioids [13].

Due to these controversies, dose threshold recommendations have limited use in clinical practice. An alternative approach would instead involve evaluating individualised risks and benefits of opioid therapy any time a change is considered. For example, a patient with knee osteoarthritis who can’t tolerate NSAIDs might be treated with a low dose of opioids, to avoid tolerance or withdrawal symptoms on discontinuation. Any consideration of a dose increase would result in re-evaluation of the patient’s pain condition, along with other opioid stewardship measures, including evaluating for opioid use disorder.

### Patients on legacy opioids

Arguably the most challenging topic in opioid management is caring for patients who have already been on long-term opioid therapy for pain management (i.e. “legacy opioids”). From 1990 to 2015, innumerable patients were initiated on long-term, and often high-dose, opioid therapy based on guidance from pharmaceutical companies, medical organisations, and regulatory authorities that recommended aggressive use of opioids for any pain conditions [14]. Longitudinal exposure to opioids results in multiple physiologic and psychological changes that create both risks and potential benefits to tapering or discontinuing opioid therapy [15,16].

As opioid dose is increased, the development of physical opioid dependence is expected [17]. However, the interaction of this process and chronic pain conditions can result in a controversial phenomenon that has been called complex persistent opioid dependence (CPOD) [18]. CPOD is a condition previously understood as a biologically distinct state between opioid dependence and opioid use disorder that is characterised by worsening pain, worsening functional status, disordered sleep, psychiatric symptoms, and fluctuations in pain and affect [19]. While tapering opioids may seem to be an appropriate response, symptoms will often paradoxically worsen, and can persist for extended periods after opioids are discontinued [15]. CPOD symptoms tend to be resistant to non-opioid and non-pharmacologic therapies, but rapidly relieved by reinstatement of opioid therapy [19]. There may be little to no distinction between CPOD and mild to moderate opioid use disorder, suggesting a prominent role for medications such as buprenorphine [18].

In addition to worsening pain, function, and psychiatric symptoms, tapering and discontinuing opioid therapy for chronic pain has been associated with several concerning outcomes among both publicly and commercially-insured populations. A study of 600 publicly-insured patients receiving long-term opioid therapy for chronic pain found that cessation of therapy was associated with increased non-prescribed heroin and other opioid use [20]. A study of Medicaid patients for whom opioids were discontinued in Vermont found that 49% had a subsequent emergency department visit or hospitalisation for opioid-related complaints; the likelihood of such admissions declined by 1% with each day the taper was extended [21]. A national study including both publicly and commercially-insured patients found a markedly higher incidence of both overdose events and mental health crises among patients in whom opioids were tapered [22]. A study of 572 patients in Seattle, Washington, found that the risk of overdose mortality was nearly 3 times higher among patients after opioid therapy was discontinued compared to those for whom opioid therapy was continued [23]. Finally, a well-controlled analysis of 1,394,102 patients in the Veterans Administration found an elevated risk of death from both overdose and suicide after cessation of opioid treatment, with greater risk the longer patients had been treated prior to cessation [24]. Outcomes appear to be worse among those patients most likely to be tapered – i.e. those with mental health or substance use disorders [21].

When pain clinics close or when clinicians treating patients with opioids relocate or retire, patients may encounter particularly stark challenges. Clinicians may “inherit” these patients, who are already being treated with opioids. The new clinicians might not have elected to prescribe opioids for those conditions, let alone at high doses. The new clinicians also might have implemented opioid stewardship measures, such as checking urine drug screens, that were not done by the former clinician. The new clinicians are thus put in the difficult position of being asked to continue care with which they do not agree, and which could put their licence at risk. In fact, some clinicians may even refuse to see these patients, as demonstrated in a survey of primary care clinics in Michigan, 40.7% of which stated their clinicians would not accept new patients receiving opioid therapy for pain [25].

We recommend the following practices for clinicians caring for patients already treated with long-term opioid therapy to balance patient-centered care and safeguard their licence. In every patient scenario, the clinician should discuss their usual opioid prescribing practices with the patient, while noting that every attempt will be made to provide continuity in care with slow adjustments to the new style of practice.

#### Contact former clinician

First, the new clinician should attempt to contact former clinicians to obtain not just medical records, but also a verbal discussion of the trajectory of the patient’s opioid treatment (e.g. was the clinician increasing, maintaining, or decreasing the dose; had the clinician discussed tapering with the patient; etc.). If the former clinician had been discussing a taper with the patient, then the new clinician could continue that discussion without it seeming to be a radical divergence from the care the patient was receiving previously. In contrast, if the former clinician was increasing the opioid dose, the new clinician might opt to continue the current dose without further increase.

#### Provide bridging opioid therapy

Second, the new clinician should refill the patient’s current opioid therapy unless there are clear reasons not to do so. The patient just transferred care and may fear losing access to opioids that they have relied upon, often for many years. The worry of losing access to opioids may be causing severe psychological distress for the patient, if not outright withdrawal symptoms in the event of a lapse in the prescription [26]. In some cases, patients have felt forced to access opioids from friends or family to bridge them to care with a new clinician, notwithstanding the knowledge that a urine drug screen would reveal non-prescribed opioid use [26]. This tension can be substantially relieved if the patient knows that they will receive a prescription for opioids at the end of the visit. This decision can be difficult for a new clinician due to concerns about a lack of benefit from long-term opioids for many conditions. However, maintaining continuity of care while transitioning to a new clinician is often more important to the patient’s well-being and safety. Adjusting to a more evidence-based pain management regimen can be achieved over time.

Some clinicians opt to issue initial prescriptions for a short period of time, such as one week. This may be reasonable if visits can be arranged, the patient has the resources to attend such frequent visits, and the insurance company will accept multiple opioid prescriptions in a 30-day period. However, this combination of factors is rare and may result in patients losing access to the medication and suffering complications including opioid withdrawal. While a strong patient-clinician relationship is essential prior to initiating an opioid taper, there are some rare circumstances (e.g. a recent overdose on the medications) that may warrant beginning a taper on the initial visit.

#### Develop a patient-centered plan

**Figure 2.**
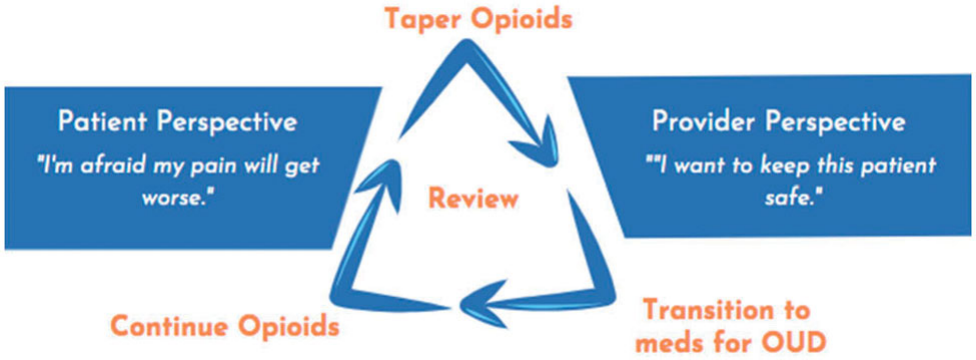
Opioid Management Options. Adapted from the authors’ public domain document “A Guide for Primary Care Providers”, San Francisco Department of Public Health, accessed at www.ciaosf.org on 30 November 2021.

Third, the new clinician should develop a patient-centered plan for opioid management. Most commonly, clinicians will have three broad options: continue opioid therapy, taper opioids, or transition to medications for opioid use disorder like methadone or buprenorphine (see Figure 2). There are nuances, such as adjusting regimens to increase safety (e.g. shifting to buprenorphine formulations approved for chronic pain). The patient may be worried about losing access to opioids, while the clinician may be worried about keeping the patient safe, but also feel pressured by peers, clinic leadership, payors, and regulators to reduce or stop prescribing opioids. In navigating the interplay between all these factors for each individual patient, frequent reassessment of dose along with the risks and benefits of opioids is necessary. In doing so, it is essential that clinicians recognise the power dynamics, avoid judging the patient, anticipate stress and possible conflicts, and empower the patient to participate in treatment planning.

It is also critical for clinicians to consider how opioids, or the loss of access to opioids, may affect the patient. Review of non-opioid pain management strategies, and the ability of the patient to access such services (e.g. access to transportation to attend physical therapy), should be conducted for all patients. Screening for mental health disorders should be conducted and referrals or treatment initiated. The patient’s social support structure should be considered, as changes in opioid therapy are more likely to be successful if the patient has sufficient social support, which can be augmented by support groups [27]. Likewise, housing and domestic dynamics should be considered. In some cases, patients may face intimate partner violence related to opioids, or may rely upon opioid diversion for financial support or even to sustain housing. While none of these justify continuing opioid prescriptions, recognition of the pressures on the patient can allow for a more open and honest dialogue regarding changes. Moreover, clinicians may wish to address these issues (e.g. initiate efforts to find more stable housing) to limit the consequences of changing opioid management.

#### Consider treatment of opioid use disorder

Fourth, clinicians should evaluate for opioid use disorder (OUD) with the Diagnostic and Statistical Manual of Psychiatric Disorders (DSM-5) criteria (of note, these criteria do not include tolerance or withdrawal when the substance of concern is prescribed). If the patient has evidence of OUD, clinicians should begin to discuss management options immediately. Transitioning a patient from long-term opioid therapy for chronic pain to treatment for OUD can be difficult for a patient, particularly when they are starting with a new clinician who they may not yet trust. Patients may justifiably feel stigmatised by a new OUD diagnosis and may require time to adjust to new plans of care. By starting this process right away, rather than waiting until they are forced to make the change, clinicians can provide some time for this transition.

Medications for OUD (MOUDs) are effective, life-saving, and always indicated for OUD, particularly moderate-to-severe disease. Of the three FDA-approved MOUDs, buprenorphine has been demonstrated to be highly effective in managing co-morbid chronic pain and OUD, with lower pain scores and higher quality of life when compared to full agonist opioids [28]. In addition, buprenorphine is inexpensive and can be dispensed from pharmacies like other medications. In contrast, methadone for OUD requires management from an opioid treatment program, which may be a barrier for some patients but helpful for others; whereas extended-release naltrexone (XR-NTX) does not provide analgesic benefits, is rarely selected by patients, has a high cost, and is complicated to administer.

#### Document pain and opioid management

Finally, clinicians should document the patient’s condition and the management plan. Comprehensive documentation would include pain history, current pain, current therapy, risk evaluation, opioid stewardship measures including screening for OUD, and plan. Pain history can be described with the “OLDCARTS” mnemonic (onset, location, duration, characterisation, aggravating and relieving factors, treatment history, and severity). In addition, clinicians should review any prior relevant labs (e.g. inflammatory markers, serologies), imaging, prior clinician notes, and any history of other non-opioid treatments. Current pain assessment should include more than the much-maligned “numeric pain scale” (e.g. the 3-item PEG scale that assesses pain intensity and interference [29,30]. Opioid stewardship measures include checking the controlled substance monitoring program (CSMP) and reporting results (e.g. “no unexpected results”), completing a consent or controlled substance agreement form, conducting urine drug screening, and providing a prescription for naloxone. Screening for OUD is important at the initial visit and then at a frequency based on the risk evaluation. The plan should include justification for continuing opioid therapy. Rationale may be based on unique elements of the pain condition, but may also be attributed to the recent transfer of the patient’s care, the need to develop a stronger patient-clinician relationship, concerns about risks of stopping therapy, or other changes to therapy recently implemented.

Documentation serves two purposes. First, patient care is more organised and systematic when documented in this manner. Second, the clinician achieves some protection from investigation into their care decisions. Reviewing publicly-available data for 118 administrative actions reported by the Medical Board of California against clinicians for prescribing controlled substances between 2017 and 2021, we found that 36% of cases were filed based on insufficient documentation, 53% documentation and negligence, and 10% negligence alone. Regulators utilise documentation as a mechanism to address prescribing practice issues, mitigate risk, and ensure appropriate treatment rationale. Including documentation of pain, stewardship measures, and justification for treatment plans can thus protect both patients and clinicians.

### Tapering opioids

Before making a decision to taper opioids, it is important to get to know the patient and their stressors, as described above. Clinicians should have already asked the patient about their perceived risks and benefits of opioid therapy, and reviewed mental health and social barriers to a successful taper. Risks related to an opioid taper include worsening pain, use of non-prescribed opioids, overdose due to reduced tolerance, and even suicide. Risks should be discussed openly with the patient. Benefits of a successful taper should be discussed, including similar or improved pain and reduced burden of and stigma related to taking opioid medications [16]. Tapering should almost never result in withdrawal symptoms, however the aetiology of opioid withdrawal is complex. If the patient or clinician is concerned about withdrawal or the patient has a history of experiencing opioid withdrawal symptoms, medications can be provided (e.g. clonidine for sweats and feeling jittery, hydroxyzine for anxiety and insomnia, ondansetron for nausea and vomiting, loperamide for diarrhoea, and NSAIDs or acetaminophen for body aches).

The clinician should then empower the patient as much as possible by allowing them to direct the nature and speed of the taper. If a patient is on multiple high-risk medications, such as benzodiazepines and more than one type of opioid, the clinician might ask the patient which medication they would like to taper first, and at what rate. This gives the patient some investment in the process and allows them to start with the medication that they feel is less critical to their well-being. A patient-directed taper may take a long time, from months to years, and have pauses along the way. Ultimately, a successful taper will look different for each patient, and may not always reach the arbitrary MME values often put forth in regulatory documents and clinical guidelines.

The most common rate of an opioid taper is a 5–20% monthly reduction from the original total daily opioid dose [31]. For example, a patient taking morphine sustained release (MSR) 90 mg three times daily (total 270 MME) might reduce to MSR 75 mg three times daily (total 225 MME, 16% reduction) in the first month. If the taper proceeded to discontinuation without a pause, then this process would take 7 months. However, pauses are likely and this taper could take much longer. Alternatively, a slower taper would be 2–10% per month (e.g. the same patient would continue to take 90 mg in the morning and at night, but reduce to 75 mg for the midday dose [a 5% reduction]; this taper would bring the patient to 45 mg three times daily [total 135 MME] after 9 months). Taper plans can be complicated and patients will benefit from seeing the taper in writing.

More rapid tapers are discouraged, unless the patient was already on a very low starting dose of opioids and is not expected to have developed tolerance or to experience withdrawal symptoms on discontinuation. A weekly reduction of 10–20% from the original dose would be considered a rapid taper, with a daily reduction of a similar amount used for a very rapid taper. Tapering opioids at this rate is dangerous and discouraged, except under very specific circumstances and with close clinical supervision.

## Opioid stewardship

Measures to improve the safety of opioid prescribing have been increasingly implemented over the past two decades. While guidelines often recommend or require clinicians to implement these strategies, evidence to suggest clinical benefits is sparse. Following opioid stewardship guidelines remains important, however, to both ensure organised opioid prescribing and to protect a clinician’s ability to practice medicine.

Opioid stewardship measures can roughly be divided into assessments, completing controlled substance agreements, checking the CSMP, and prescribing naloxone. The frequency of these measures varies by state and often by clinic. It is important to inform patients of the need to conduct these steps whenever prescribing opioids long-term.

### Assessments

There are four main assessments of potential harms and benefits to consider when prescribing long-term opioid therapy: risk factors, pain and function, urine drug screening, and evaluating for opioid use disorder.

#### Risk factors

Many guidelines recommend assessing patients for the risk of developing problematic opioid use prior to starting long-term opioid therapy. There are three resulting questions: what assessment instrument to use, when to administer the instrument, and what to do with the results.

Many different risk factor assessments have been developed, yet have not been shown to prospectively predict subsequent problematic opioid use. Commonly-used tools in practice include the Pain Medication Questionnaire, the Opioid Risk Tool, the Brief Risk Questionnaire, the Brief Risk Interview, and the Screener and Opioid Assessment for Patients with Pain, though only the Pain Medication Questionnaire appeared partially useful in clinical practice based on a systematic review [30]. In this review, the best predictors of later problems with prescribed opioids were a history of substance use disorders (particularly OUD), mental health disorders (particularly personality disorders), and receipt of certain psychiatric medications (e.g. atypical antipsychotics). However, no tools were found useful for identifying patients at lower risk, thus even these patient characteristics should be evaluated cautiously, given the risks of further stigmatising patients. With regard to timing, this risk assessment should happen prior to initiating opioid therapy, or whenever a patient receiving opioids transfers to a new clinician. A repeat evaluation might be performed later, as mental health diagnoses or medications may change. However, the primary tool to gauge risk after opioid therapy is initiated will be evaluating for OUD. More importantly, initial screening for risk factors should not be used to determine if opioids are indicated or contraindicated, but instead to determine the intensity and frequency of opioid stewardship monitoring. In the presence of risk factors a clinician may, for example, elect to increase the frequency of assessments to ensure benefit.

#### Pain and function

Clinicians should assess pain before starting opioid treatment, within three months of starting therapy, and at least annually thereafter [4]. The most common pain assessment is the pain intensity numerical rating scale, which clinicians are required to ask patients when checking vital signs. Often referred to as the “fifth vital sign”, this assessment is not helpful for complaints of chronic pain because it neither addresses pain that is not present at the moment of the assessment, nor does it address the impact of pain on function or quality of life. A patient with debilitating pain when attempting to sleep at night might have no pain while at clinic, but feel that they must report a pain score of 10 to ensure that their pain is addressed. Clinicians may disregard reports of pain that are incongruent with patient behaviour during the visit.

The Pain, Enjoyment, and General Activity (PEG) scale is a three-question tool to assess average pain, pain interference with enjoyment of life, and pain inference with general activity over the past week [29]. By measuring average pain, this scale allows patients to report on pain that they experienced outside of the clinic visit. Even more critical, by addressing enjoyment of life and function, the PEG incorporates quality of life and function into a single simple questionnaire. Helping patients set functional goals focussing on their activities of daily living can both empower the patient and give the clinician a tool for treatment planning; if function is not improving, the PEG can provide objective evidence to initiate a change in pain management strategies, like tapering and switching to a different medication. The PEG has been demonstrated to be as valid and reliable as the much longer Brief Pain Inventory and sensitive to changes in pain over time [29]. We recommend using the PEG at each visit, and sharing results with patients in order to demonstrate evidence of benefit or lack thereof from recent interventions.

#### Urine drug screening

Most guidelines recommend urine drug screening (UDS) for patients who are prescribed opioids long-term for any indication at least annually [4]. Many clinics adopt uniform UDS frequency in order to avoid unintentional bias, such as more frequent UDS for minority patients. UDS results indicate substances and associated metabolites present within a timeframe of use. Interpreting and applying these results in clinical care requires some expertise or clinical support.

##### Interpreting urine drug screens

**Figure 3.**
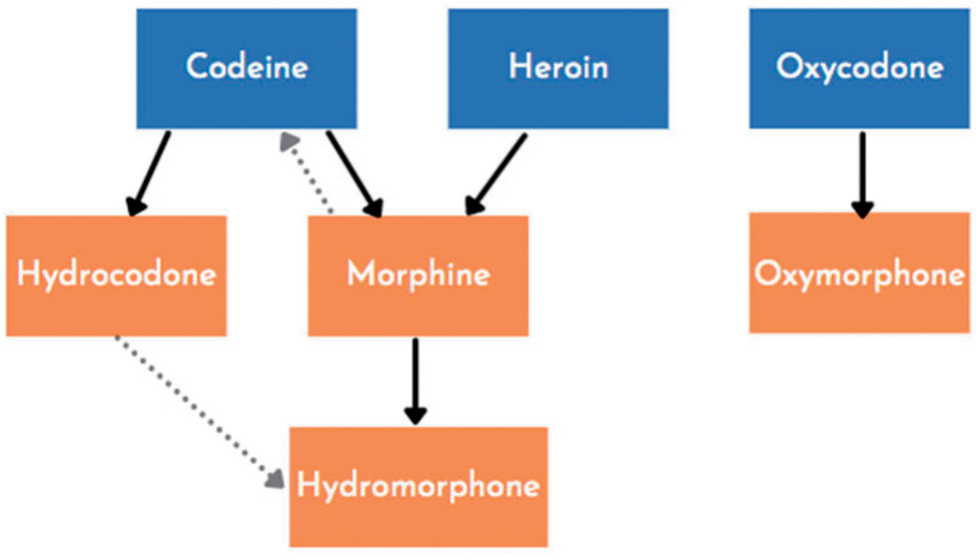
Simplified opioid metabolic pathways. Adapted from the authors’ public domain document “A Guide for Primary Care Providers”, San Francisco Department of Public Health, accessed at www.ciaosf.org on 30 November 2021. Note: buprenorphine, fentanyl, and methadone all require a separate test.

Clinics often utilise UDS point-of-care assays, with qualitative results and significant false positive and negative rates. These assays do not test for all substances; methadone, buprenorphine, and fentanyl usually require separate tests. A basic understanding of opioid metabolism is essential to interpret UDS results (see Figure 3). This information can help a clinician avoid incorrectly accusing a patient of taking non-prescribed opioids based on UDS results. Clinicians should check with the laboratory where the assay is performed to determine any unique aspects to that particular assay. The laboratory may be able to preform confirmatory testing using gas chromatography/mass spectrometry (GC/MS) or liquid chromatography/mass spectrometry (LC-MS). Stigma-free language should be used to describe UDS results, with terms like “expected” and “unexpected” results rather than “clean” or “dirty” urine.

##### Use of urine drug screens

The most important step a clinician can make after receiving discordant UDS results is to speak with the patient in a non-judgmental manner. The clinician can assess if the patient is taking the therapy. In cases of low-dose or intermittent therapy, the patient may only take the opioid when pain is severe, and thus would not necessarily be expected to have a UDS positive for the prescribed opioid. Sometimes, hydration or body mass can obscure low to moderate dose opioids from UDS detection. UDS does not establish an OUD diagnosis, nor are UDS results even a criterion for OUD. After discussing results with the patient, a clinician may decide to repeat OUD screening or discuss concerns of medication diversion with the patient. These are not easy conversations and they do not always result in mutually agreeable conclusions.

Some clinic systems use non-opioid results from UDS, such as evidence of cannabis, cocaine, or methamphetamine use, to determine whether or not opioid therapy will be continued. There is a great controversy regarding this approach. First, there are data to support cannabis use as a potential pain management agent. Cannabis use among people who use drugs and suffer from chronic pain has been associated with less non-prescribed opioid use [32]. Stimulants, while not recommended for pain management, are used by some patients for that purpose [33]. Stimulant use should thus prompt a clinician to identify the reasons for use. Clinicians may wish to use the identification of other substances in the UDS as an opportunity to explore the patient’s pain and pain management practices, as well as implementing strategies to minimise risks associated with use of those substances. If UDS results are repeatedly positive for concerning substances, and OUD or diversion of medications is suspected, then therapy will typically require change.

#### Evaluating for opioid use disorder

Utilising the DSM-5 criteria to evaluate for OUD is an important step in management of long-term opioid therapy in a patient with chronic pain. There are 11 criteria for OUD, four of which can be considered patterns of use (using more or for longer than intended, being unable to stop or cut down, spending excessive time dealing with the substance, and craving) and five of which are related to continued use in the presence of known problems (unfulfilled responsibilities, social and interpersonal problems, reduced activities, physical hazards, and health problems caused by the substance). The final two criteria are the development of tolerance and symptoms of withdrawal. However, these criteria do not apply when a patient is prescribed the substance because physiologic effects are expected. Severity of OUD is graded as mild if the patient meets 2–3 criteria, moderate for 4–5 criteria, and severe for 6 or more criteria. Criteria should be present for six months or longer, which offers a window of opportunity for a new clinician to form a working relationship with patients before confirming an OUD diagnosis and shifting to OUD treatment.

A patient receiving long-term opioid therapy for chronic pain may be initially resistant to a diagnosis of OUD. We recommend discussing findings with the patient and revisiting the new diagnosis over several visits. It can be helpful to focus the conversation on safety, risks, and benefits of opioids, including treatment options like buprenorphine for continued pain management with simultaneous risk reduction. Transitioning from accepting use of opioids as a treatment for chronic pain to treatment for a use disorder can take time. Nonetheless, prioritising transparency and engaging the patient from the moment of diagnosis is valuable to build trust in the patient-clinician relationship

### Controlled substance agreements

Most guidelines recommend some form of a controlled substance agreement for patients who are prescribed opioids long-term for chronic pain [4,34]. Several studies and systematic reviews have found no evidence that controlled substance agreements are associated with lower rates of problematic opioid use [35,36]. A study of patients receiving opioids for chronic pain found that patients were not consistently aware they had a controlled substance agreement [37]. Another study of 430 clinicians across the United States who used controlled substance agreements in their practices found those agreements to be time consuming, minimally effective, generally written far above appropriate reading levels, and serving primarily to convey the consequences of non-compliance [38]. Two-thirds of respondents felt agreements were “worth the effort,” yet only 28% felt they played a role in reducing misuse of opioids. Since many guidelines recommend these agreements [39] and they often serve as an opportunity to discuss risks and as a proxy for informed consent, we generally recommend that clinicians at a minimum provide written information to patients about the risks and benefits of opioid therapy and document patient understanding and agreement.

Of note, we refer to these agreements as controlled substance agreements, rather than “pain agreements”, because they are utilised only when a patient is prescribed controlled substances (patients are not required to sign an agreement in order to access NSAIDs or physical therapy for painful conditions).

### Controlled substance monitoring programs

Most guidelines and state laws require clinicians to check CSMPs on a periodic basis, with varying frequency, when prescribing opioids [4,34]. We refer to these systems as CSMPs rather than “prescription drug monitoring programs” or “PDMPs” because nearly all of them include only controlled substances. Moreover, most CSMPs are controlled by justice departments rather than healthcare agencies, which leads to a service that is oriented towards investigations and punitive actions rather than healthcare and patient support. This has resulted in multiple concerns regarding patient privacy, public health goals, and the use of CSMP data [40].

Studies generally show an association between CSMP implementation and reduced prescribing of certain opioids, yet often in confounding patterns [41]. The presence of a legal mandate to use the CSMP appears to be necessary to consistently reduce opioid prescribing, and is now present in many states. However, the effect of CSMPs on opioid-related mortality is mixed, with some studies showing a reduction in mortality and others an increase [42]. Studies showing a reduction in mortality often focus on specific prescribed opioids, while studies suggesting an increase in mortality tend to focus on overall opioid overdose. Moreover, concerns have been raised regarding the effect of CSMPs on clinical decision-making, such as increased prescribing of less-regulated sedating substances (e.g. gabapentin) or summarily discharging patients from care to – in clinicians’ own words – “scrub out” “deceptive” patients [43]. These findings lead to the question of how one is to use CSMPs to generate benefit. Much like UDS, we recommend speaking with patients openly about any concerning CSMP results (e.g. receiving controlled substances from multiple prescribers, filling controlled substances at frequent intervals). A patient who is transferring care, for example, may have to seek care at emergency departments in order to avert withdrawal from opioids. An open discussion with patients about these concerns will serve their care better than making assumptions about opioid misuse or diversion.

### Naloxone

Naloxone is a short-acting *mu* opioid receptor antagonist used for many decades to reverse opioid overdose. Distribution of naloxone to people who use drugs has been associated with a substantial reduction in opioid overdose mortality in communities in which it is distributed compared to communities in which it is not distributed [44]. Moreover, the reduction in mortality appears to be dose-dependent, such that the more naloxone that is distributed, the greater the relative reduction in mortality. Numerous studies have also demonstrated that naloxone distribution is safe, results in sufficient transfer of knowledge, and improves skills in responding to overdose [45]. Distribution of naloxone has become common throughout the United States and many other countries.

Co-prescription of naloxone with opioids is distinct from other efforts to distribute naloxone. The CDC recommends, and many states have passed legislation mandating, co-prescription of naloxone under certain conditions [4]. The 2016 CDC guidelines suggest co-prescribing naloxone in the presence of an opioid dose greater than 50 MME, concomitant benzodiazepines, the presence of any substance use disorder, a history of opioid overdose, or any other risk factor for overdose. We would broaden that guidance to include prescribing naloxone for anyone who uses any non-prescribed opioids or, in the setting of fentanyl contamination of other drug supplies, anyone who uses non-prescribed drugs of any kind. Many states also explicitly authorise prescription of naloxone to a third party (i.e. prescription of naloxone to a family member) [46]. Instructing patients or their caregivers in when and how to use naloxone is also a good opportunity to discuss the risks of opioid medications.

The data to support co-prescription of naloxone to patients receiving long-term opioid therapy are limited. Multiple studies have evaluated the impact of educational initiatives to increase naloxone co-prescribing [47–52]. However, only one study provides any outcome data for patients co-prescribed naloxone [53]. This study was conducted from 2013–2015 utilising the off-label nasal naloxone product that required extensive assembly. Clinicians in a clinic system in San Francisco were advised to offer naloxone to all patients receiving opioid prescriptions. Pharmacists were instructed on how to order and dispense the off-label formulation of naloxone. Overall, 38% of patients prescribed long-term opioid therapy were prescribed naloxone. Patients who received naloxone had 63% fewer opioid-related emergency department visits in the year after receipt, compared to patients who did not receive naloxone. One opioid-related emergency department visit was prevented for each 29 patients prescribed naloxone.

From the same study, 60 naloxone recipients were interviewed. Nearly half of the patients who reported having experienced an opioid-related event involving respiratory arrest and the need for assistance to be woken up denied having an “overdose.” Moreover, only 10% had ever accessed naloxone from another source (e.g. syringe access program), suggesting that co-prescribed naloxone reaches a different population than community distribution [54]. Clinicians should use non-confrontational language when discussing naloxone with patients, such as referring to overdose as a “bad reaction” and to naloxone as an “antidote”).

There have been three major formulations of naloxone on the market in recent years: intramuscular (IM) by vial and syringe, IM by autoinjector, and intranasal. The autoinjector is no longer available and the IM vial and syringe is generally reserved for distribution programs. The vast majority of naloxone co-prescribed with opioids is the intranasal formulation. This product was developed for lay usage and thus comes pre-packaged with patient information that obviates the need for clinicians to separately educate patients on overdose response.

### Documentation

As described in “Patients on legacy opioids” , documentation of opioid stewardship efforts is essential to both protect patient safety and reduce clinician liability. Clinicians may wish to develop a “smart phrase” or other problem list template for patients prescribed long-term opioid therapy that addresses pain, function, stewardship, and therapeutic plan with rationale. By capturing these data in medical records, clinicians are better able to closely track the well-being of their patients while also defending their medical decisions in the event of oversight.

## Managing opioid use disorder

Every clinician serving patients on long-term opioid therapy for chronic pain should be prepared to manage OUD. Screening for OUD, addressed above, should be performed periodically in all patients managed with long-term opioid therapy. Evidence of OUD should prompt consideration of MOUD. If a clinician is not directly offering OUD treatment, a warm handoff to other clinicians is critical; handing a patient a list of programs to call on their own is insufficient.

### Medications for OUD

MOUD, including methadone, buprenorphine, and XR-NTX, provide the greatest benefit for patients with OUD. All three medications have been demonstrated to reduce non-prescribed opioid use. Methadone and buprenorphine, but not XR-NTX, have also been shown to reduce overdose mortality [55,56] and to be safe and effective during pregnancy [57]. Methadone and buprenorphine have also been shown to have a lower risk of opioid overdose mortality when compared to abstinence-based treatments for OUD; this is likely because abstinence-based approaches result in reduced tolerance and greater risk for overdose when patients relapse to non-prescribed opioid use [58]. OUD is a chronic disease and will often require many years, or even a lifetime, of treatment. Thus, MOUD should be considered a long-term therapy, akin to insulin treatment for diabetes. Treatment should not be discontinued unless there is no benefit to the patient.

**Figure 4.**
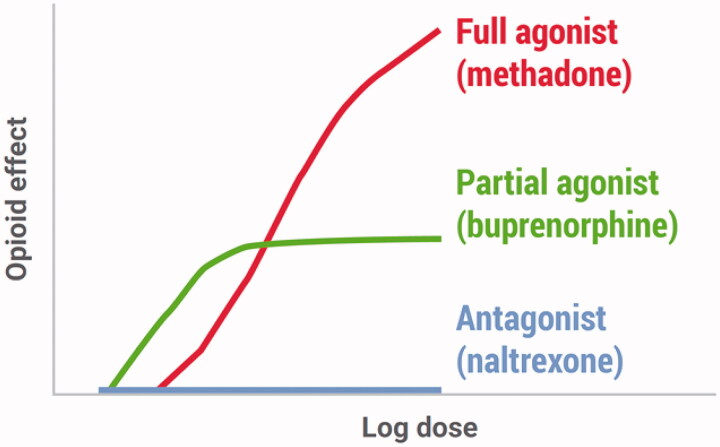
*Mu* opioid receptor activities of medications used for opioid use disorder. (Only the extended-release formulation of naltrexone is approved for for opioid use disorder (specifically for prevention of relapse to opioid dependence)). Figure taken with permission from the authors’ public domain document “A Guide for Primary Care Providers”, San Francisco Department of Public Health, accessed at www.ciaosf.org on 30 November 2021.

MOUD have been reviewed extensively [59]. Briefly, methadone is a full agonist at the *mu* opioid receptor, approved by the FDA for analgesic and antitussive use in 1947 and for treatment of OUD in 1972 (see Figure 4) [60]. Methadone is a DEA schedule II medication that can only be provided for OUD in dedicated opioid treatment programs (OTPs) that are approved for methadone maintenance. Buprenorphine is a partial agonist with high affinity for the *mu* opioid receptor, such that other opioids have little to no effect. Buprenorphine was initially approved by the FDA for analgesia in 1985, then for OUD in 2002 alongside the DATA 2000 subsection of the Children’s Health Act that authorised treatment of OUD outside of dedicated treatment programs. Buprenorphine is a DEA schedule III medication and can be prescribed by any clinician for pain management in outpatient settings, although it requires a DATA 2000 waiver when used to treat OUD. XR-NTX, a *mu* opioid receptor antagonist, was FDA-approved for treatment of alcohol use disorder in 2006 and relapse to OUD after detoxification in 2010. XR-NTX is not a controlled substance and can be prescribed by any clinician, although obtaining the product and administering the injection may be difficult for clinicians who do not do so regularly [61] and few patients select this option [62]. Although oral naltrexone is sometimes used, it is not FDA-approved for OUD and is associated with a high rate of opioid overdose [63,64].

### Buprenorphine

Buprenorphine is a powerful analgesic that treats OUD with minimal risk of respiratory depression and can be easily provided in primary care settings. These benefits help to reduce the stigma of OUD treatment [65]. Patients transitioned from opioid therapy for chronic pain often select this medication for OUD treatment. A study of 35 patients with OUD treated with 200–1370 MME for chronic pain and then transitioned to buprenorphine found a mean reduction in pain scores from 7.2 to 3.5 (*p* < 0.001) and an increase in quality-of-life scores from 6.1 to 7.1 (*p* = 0.005) [28]. While not all patients with chronic pain who have been treated with full agonist opioids will have better outcomes with buprenorphine, we generally recommend a trial for patients with co-morbid OUD. In addition, a systematic review has shown buprenorphine to be modestly beneficial in reducing pain intensity even for patients without OUD [66].

#### Obtaining a waiver to prescribe buprenorphine for OUD

The procedure for obtaining a DATA 2000 waiver was simplified in 2021. Clinicians now need only request a waiver on the Substance Abuse and Mental Health Services Administration (SAMHSA) website. The request will take approximately 2–4 weeks to process, after which the clinician will receive a new DEA registration including their “X number” that can be used for treating patients with buprenorphine for OUD. If a clinician wishes to treat more than 30 patients at any one time with buprenorphine, there are additional requirements. First, the clinician must complete a training (8 h for MDs or DOs; 24 h for NPs, PAs, CNMs, CNSs, and CNRAs) and submit a request to SAMHSA to treat up to 100 persons. After another year, MDs and DOs can submit a subsequent request to SAMHSA to treat up to 275 patients at a time. There are exceptions to some of these requirements, such as being board-certified in addiction medicine. The DATA 2000 waiver and associated “X number” are free and do not expire, but are linked to an active DEA registration.

#### Planning for buprenorphine

As noted above, patients may require time to adjust to a new diagnosis of OUD. After introducing the concept of treatment for OUD, clinicians should describe the three available medications and offer to assist the patient in receiving whichever treatment they prefer. This can be a good time to talk about the details of buprenorphine.

First, buprenorphine for OUD is available mono-formulated or co-formulated with naloxone, in tablets, films, and an injection. The co-formulated product was created to alleviate DEA concerns of diversion or injection of the product. Naloxone has minimal bioavailability when used sublingually, but reduces the modest euphoric effect of buprenorphine when the co-formulated product is injected [67]. Any of these formulations can be used to treat OUD and the safety of buprenorphine is independent of the presence of naloxone. The co-formulated product is generally recommended for first-line OUD treatment in order to reduce the risk that a patient may inject the product. However, some patients report side effects, such as a headache or flank pain, which are often due to the naloxone component, and may have better results with the mono-formulated product [68]. Sublingual films can be cut into smaller doses and tablets can be divided along scores; these should be left under the tongue until they are completely dissolved because they will not be properly absorbed if swallowed. The extended-release injection is a mono-formulated product. Treatment should be considered long-term and potentially lifelong, although after not using non-prescribed opioids for years, some patients do well on minimal doses of buprenorphine.

Second, when starting buprenorphine therapy, there is a risk for withdrawal symptoms. Traditional initiation of therapy, in fact, requires the patient to be in withdrawal, for which clinicians may offer patients medications to self-manage. Precipitated withdrawal, which can occur during buprenorphine initiation, involves symptoms of severe opioid withdrawal that may require emergency medical care [69]. This condition has become more frequent in the context of fentanyl use, leading to novel approaches to initiating buprenorphine like overlap or low-dose initiation (see “Initiating Buprenorphine”) [70].

Third, there are no absolute contraindications to buprenorphine treatment. Buprenorphine is proven to be safe and effective during pregnancy, although the mono-formulated buprenorphine is recommended over the co-formulated buprenorphine-naloxone simply because there is no therapeutic value to the naloxone component [57,71]. The mono-formulated product is also recommended for patients with significant liver disease due to poor hepatic metabolism of naloxone. While buprenorphine has a markedly low risk for respiratory depression due to the ceiling effect, the addition of benzodiazepines or alcohol can increase that risk. However, for patients already using depressants, the transition from full agonist opioids to buprenorphine is associated with a substantial reduction in the risk of opioid overdose. In a case-crossover study analysing a dataset of 14 million person-days for 23,036 persons who experienced drug poisoning events, high-dose benzodiazepine use was associated with increased poisoning events in combination with buprenorphine, but this was significantly lower than the risk of poisoning when benzodiazepines were used without buprenorphine [72]. The FDA and SAMHSA have clearly stated that use of sedatives or hypnotics is not a contraindication to treatment with buprenorphine [73]. While a clinician should continue to address the use of benzodiazepines or alcohol in a patient prescribed buprenorphine, such use should not impede buprenorphine therapy.

Fourth, buprenorphine is commonly provided with therapy such as counselling, cognitive behavioural therapy, or support groups. Previously, SAMHSA required clinicians to confirm they had access to counselling services prior to obtaining a DATA 2000 waiver; this requirement was lifted in 2021. A randomised controlled trial of buprenorphine treatment with and without counselling found no difference in the effectiveness of treatment, measured as days of non-prescribed opioid use over 6 months of follow-up [74]. While counselling may be beneficial, particularly for patients with comorbid disorders such as post-traumatic stress disorder [75], the absence of counselling availability or patient unwillingness to engage in counselling should not be barriers to buprenorphine treatment [76].

#### Initiating buprenorphine

While buprenorphine can now be provided entirely through telehealth [77], the standard way to initiate buprenorphine in an outpatient setting is to first visit with the patient to review the plan and issue a prescription of buprenorphine covering approximately one week of therapy for the patient to pick up at the pharmacy. The patient may sign a consent form for buprenorphine treatment in lieu of a controlled substance agreement. The patient should then abstain from opioids for 12 to 48 h (e.g. approximately 12 h for heroin, 48 h for methadone) in order to enter significant opioid withdrawal; their Clinical Opioid Withdrawal Scale (COWS) score should be greater than 8 [78]. The patient would then take a 4 mg dose of buprenorphine and wait 1–2 h. If they were still experiencing withdrawal, they would take another 4 mg dose of buprenorphine and repeat the same procedure. The maximum total dose on Day 1 would be 12 mg of buprenorphine. On Day 2, they would take the total dose from Day 1 (or less if they felt sedated) and increase to a total dose of 16 mg if needed to treat withdrawal. They would then continue that procedure to a maximum daily dose of 32 mg of buprenorphine if needed. This procedure is similar for inpatients, but can be done more rapidly given the close oversight (i.e. up to 16 mg on Day 1 and 32 mg on Day 2). More details on buprenorphine initiation can be found in the resource, SAMHSA Treatment Improvement Protocol 63 [79].

Initiating buprenorphine originally was done in clinics, which was a major barrier to clinicians utilising this therapy. Data from New York City demonstrated that outcomes were similar when initiating buprenorphine at home or in a clinic [80]. Since that time, home initiation has become common practice.

Unfortunately, the replacement of heroin and other opioids on the street with illicitly-manufactured fentanyl has complicated buprenorphine initiation. Due to its extreme potency and storage in fatty tissues, patients develop an extremely high tolerance to fentanyl, which may not be sufficiently reversed by a period of abstinence. Thus, even if a patient is in significant withdrawal, administration of buprenorphine in a standard initiation protocol can result in precipitated withdrawal [70,81]. This has led to the emergence of “overlap initiation” (also referred to as “low dose buprenorphine initiation” or “microdosing”) protocols that allow for buprenorphine to be started while a person continues to use full agonist opioids without the need to develop opioid withdrawal [82]. This approach to starting buprenorphine, while a patient continues to take full agonist opioids, is quickly becoming standard practice in many areas, ahead of needed research.

#### Continuing buprenorphine

Once a patient has reached a stable dose of buprenorphine, visit frequency is based on stability and response to treatment. Clinicians should discuss with the patient buprenorphine adherence, non-prescribed opioid use, healthcare maintenance, mental health needs, expected monitoring measure such as UDS and CSMP review, and other SUD assessments. After a period of closer observation, patients who are adherent to buprenorphine and cease non-prescribed opioid use might be seen just at intervals needed for their other medical complaints. In contrast, a patient who is struggling with either of those metrics would warrant more frequent visits. If buprenorphine treatment is unsuccessful at reducing non-prescribed opioid use, an alternative medication (i.e. methadone or XR-NTX) may be appropriate. However, if the patient remains adherent to buprenorphine, a clinician may opt to continue the medication if they feel there is a substantial reduction in the risk of opioid overdose due to the high affinity of buprenorphine to *mu* opioid receptors.

Buprenorphine treatment is intended to provide patients with the ability to control their opioid use. It is common that some patients find it difficult to entirely forego non-prescribed opioid use. Such cases should not be addressed punitively but rather therapeutically within the patient-clinician relationship or by a referral to a higher level of care. Some patients may be empowered to control their opioid use by a frank discussion of the role of UDS in measuring the effectiveness of buprenorphine treatment (i.e. by demonstrating an absence of non-prescribed opioids, a patient demonstrates that they are able to control their non-prescribed opioid use). As buprenorphine does not treat other SUDs, ongoing use of other substances (e.g. cocaine or methamphetamine) should not affect the decision to continue buprenorphine treatment.

#### Buprenorphine for pain

Buprenorphine can also be prescribed for chronic pain, although obtaining insurance coverage for high-dose buprenorphine when treating chronic pain in the absence of OUD may be difficult. The transdermal patch, a low-dose option, is only intended for pain and not OUD. While any formulation can be used off-label for chronic pain, the sublingual and subcutaneous formulations are approved only for OUD treatment and the buccal and transdermal formulations are the two that have demonstrated efficacy for pain [83]. When prescribing buprenorphine exclusively for pain without OUD, there is no need for a DATA 2000 waiver and the standard DEA registration number can be used. Buprenorphine is generally administered two to three times daily in this scenario to maximise analgesic benefits and can be dosed with this schedule for patients being treated for concurrent pain and OUD.

An additional issue arises when a patient taking buprenorphine experiences acute pain or requires surgery. In most of these cases, buprenorphine dose can be increased in both dose and frequency. In addition, non-opioid analgesics should be used along with local nerve blocks. Fentanyl or hydromorphone administered at relatively high doses may also be used, alongside buprenorphine, to maximise opioid effects. A multi-disciplinary expert panel now agrees that buprenorphine should not be routinely discontinued during the peri-operative period, as it can result in extreme pain and a high risk for relapse to non-prescribed opioids [84].

### Treatment options for other substance use disorders

Use of multiple substances is common among patients with OUD. Screening and offering treatment for all SUDs is critical to supporting patient health. In some cases, patients prioritise treatment of one SUD over another, and clinicians can empower patients by respecting that choice. The DSM-5 criteria for SUDs are the same across substances. Most clinicians are well-trained to identify and treat tobacco use disorder, including using nicotine replacement therapy, bupropion, and varenicline. Tobacco cessation behavioural interventions are also effective. There are three FDA-approved medications for alcohol use disorder: disulphiram, acamprosate, and immediate-release oral or extended-release injectable naltrexone. In addition, topiramate, though not FDA-approved for alcohol use disorder, has been recommended by some guidelines as first-line therapy as well [85]. Cognitive behavioural therapy, mindfulness-based therapies, and Alcoholics Anonymous may also benefit patients with alcohol use disorder. There are no FDA-approved medications for stimulant use disorders, although mirtazapine, bupropion, bupropion plus naltrexone, and methylphenidate have shown some promise for those with methamphetamine use disorder [86–88] and psychostimulants, bupropion, and topiramate have shown some promise for cocaine use disorder [89,90]. Contingency management, a behavioural tool that incentivizes patients with financial or other rewards for biological proof of drug abstinence, has demonstrated significant benefits for patients with stimulant use disorders [91,92]. While availability of contingency management has been very limited, the state of California received approval from the Centres for Medicare Services to initiate a Contingency Management Pilot program for July 2022 through March 2024, to determine how to scale this proven treatment for stimulant use disorder through Medi-Cal, the state’s public health insurance [93].

### Panel management for patients with substance use disorders

After identifying an SUD in a patient, the clinician should not only provide or arrange for treatment, but should also provide preventative services to minimise associated health sequelae. People who use non-prescribed substances, in particular those who inject drugs, should be screened at least annually for HIV, hepatitis B, hepatitis C, sexually-transmitted infections, and tuberculosis. Immunizations should be offered for hepatitis A and B, human papillomavirus, tetanus-diphtheria-pertussis, influenza, pneumococcus, and COVID-19. Patients should be educated regarding safe substance use practices, including clean injection equipment, and should receive a prescription for naloxone. Pre-exposure prophylaxis for HIV prevention should be considered, particularly for patients with high-risk sexual activity. Co-morbid psychiatric disorders should be addressed. Finally, cardiac risk factors should be aggressively addressed, particularly for patients using stimulants. Most deaths attributed to acute stimulant toxicity are related to cardiac and cerebrovascular disease, yet preventive cardiac interventions are often missed in this population [94,95]. Clinicians should consider, for example, aggressive smoking cessation in a patient who continues to use stimulants, which may lessen the progression of cardiovascular disease.

## Conclusions

There is no easy solution to managing opioids in clinical care. Prescribing opioids without oversight is too risky, yet refusing to prescribe opioids at all is inhumane and may be equally risky. Opioids remain a key part of any medical practice and should be utilised when needed. Clinicians need to take multiple considerations into account, including the pain condition being treated, treatment history, presence of mental health and use disorders, and social determinants of health, while recognising the impact of regulatory pressures on their own decision-making. The use of medications to treat opioid use disorder is critical to finding a path through the current crisis. Buprenorphine in particular has utility in primary care settings and has recently become an easier option for clinicians due to regulatory changes. Good documentation is critical to ensure not only patient safety, but also to protect clinicians attempting to balance the benefits and harms of opioids in clinical practice.

This review is not intended to replace existing guidelines or clinical judgement. Instead, we aimed to provide a perspective that complements clinical and regulatory guidelines, including a review of existing evidence and a patient-centered, practical approach to managing opioids in primary care, whether for pain management or for treatment of a use disorder. This review of literature, alongside regulatory and clinical guidance, provides a basis for developing individualised, patient-centered care for each patient who already uses opioids or may be considered for potential opioid therapy.

## Data Availability

Data sharing not applicable – no new data generated.
